# It takes time to synchronize: the emergence of dyadic heart-rate synchrony during music therapy in neurorehabilitation

**DOI:** 10.3389/fpsyg.2026.1629778

**Published:** 2026-04-17

**Authors:** Sun Sun Yap, Fabian T. Ramseyer, Jörg Fachner, Clemens Maidhof, Wolfgang Tschacher, Gerhard Tucek

**Affiliations:** 1 Department of Health Sciences, Institute of Health Sciences & Midwifery, IMC University of Applied Sciences Krems, Krems an der Donau, Austria; 2 Josef Ressel Center for Personalized Music Therapy, IMC University of Applied Sciences Krems, Krems an der Donau, Austria; 3 Cambridge Institute for Music Therapy Research, Anglia Ruskin University, Cambridge, United Kingdom; 4 Department of Neurology, University Hospital Tulln-Klosterneuburg, NOE LGA, Karl Landsteiner University, Tulln, Austria; 5 Department for Clinical Psychology and Psychotherapy, Institute of Psychology, University of Bern, Bern, Switzerland; 6 Department of Experimental Psychology, University Hospital of Psychiatry and Psychotherapy, Bern, Switzerland

**Keywords:** heart-rate synchrony, moment of interest, music therapy, neurological rehabilitation, nonverbal synchrony, therapy duration

## Abstract

Heart-rate (HR) synchrony between individuals has been linked to emotional contagion and shared experience. Research in psychotherapy has also associated this type of synchrony with therapists demonstrating more empathic and attentive behavior, as well as progress and alliance ratings. Using dyadic HR data, session videos and notes, this paper investigated the emergence of dyadic HR synchrony during music therapy (MT) interventions in a neurorehabilitation setting between 11 in-patients (mean age 51 years ± 6.48) and one music therapist (43 years) who were selected by convenience sampling. HR synchrony during moments of interest (MOI; mean duration 76 ± 20.7 s) within the MT intervention period (mean duration 25.62 ± 7.34 min), as selected by the music therapist was explored. Focus was also given to the leading characteristics during HR synchrony, as well as the relationships between HR synchrony and nonverbal (NV) synchrony before and after the MT intervention, along with the association with patients' therapy readiness. We found that dyadic HR synchrony occurred beyond chance but significant associations to the patient's therapy readiness and NV synchrony could not be demonstrated. However, there was a very strong association between HR synchrony and the duration of the MT interventions, especially the 20-25 min block, suggesting that a certain duration may be needed for HR synchrony to emerge. HR synchrony during MOI segments was not significantly higher than the MT intervention average but more than half of them overlapped with the four highest and lowest HR synchrony segments, which could suggest that MOI segments may be detected physiologically. The ability to access previously hidden dyadic physiological changes and understanding the impact of MT intervention duration on HR synchrony could have important implications for clinical practice and policies in neurorehabilitation.

## 1 Introduction

According to the Austrian Professional Association of Music Therapy, music therapy (MT) is the planned treatment of individuals with physical, mental, intellectual, and social disorders. Its goals are to alleviate symptoms, change treatment-related behaviors, promote health, and it takes place within a therapeutic relationship. MT is closely related to other disciplines, including psychotherapy, psychology, medicine, musicology, and pedagogy (Österreichischer Berufsverband der MusiktherapeutInnen, 2025). The current exploratory case-series study was an extension to a research project (Right Period Project) focusing on the chronobiological aspects of therapy in a neurorehabilitation setting at the Josef Ressel Centre for the Foundation of Personalised Music Therapy (JRZ) in Krems, Lower Austria. Having its roots in traditional Oriental MT, the tenets of the Krems approach to MT include shared moments of musical encounter, the principles of harmonizing and regulating psychological and physical states (Tucek et al., 2021). The JRZ emphasizes the importance of viewing patients as individuals rather than merely concentrating on their illnesses (Tucek et al., 2021). Its research approach is grounded in an anthropological paradigm and prioritizes personalized therapy over standardized treatment procedures. Additionally, the JRZ aims to bridge the gap between research and practical clinical applications by collecting ecologically valid data about the MT process and the lived experiences of the therapist-patient relationship (Fachner et al., 2021). This led to a focus on creating new models that aim to bring research from the laboratory to the bedside (Fachner et al., 2021; Tucek et al., 2021).

The JRZ aligns with the French model of action research developed by (Desroche 1990), which conceptualizes research as a dialogical, reflexive, and transformative process in which the practitioner simultaneously assumes the roles of actor, observer, and self-examining participant. Thus, the therapist is a legitimate researcher and a subject of inquiry within his or her own practice. This dual role does not constitute a methodological deficit; rather, it reflects an epistemological stance that recognizes subjectivity, contextual embeddedness, and experiential transformation as integral components of knowledge production. From JRZ's anthropological perspective, the human being is understood not as an isolated object but as a relational and situated entity. The therapeutic encounter thus becomes not only a site of intervention but also a site of knowledge. The therapist's embodied presence, biographical background, and situational responsiveness form part of the relational field and therefore legitimately enter the research process. The legitimacy of such self-inquiry arises from the fact that therapeutic practice cannot be fully captured from an external vantage point; it requires an internally situated perspective articulated through disciplined reflexivity. The therapist-researcher not only observes but also experiences, senses, and reflects. Lived experience within the therapeutic relationship becomes a source of insight that cannot be replaced by standardized procedures. In this sense, the therapist as a researching agent is not merely permissible but epistemically indispensable for grasping the complexity of music therapy processes and the singularity of therapeutic encounters.

The world around us seems to be fond of synchrony, a phenomenon that we often observe in our surroundings and daily lives (Strogatz and Stewart, 1993). The term “synchrony” comes from the Ancient Greek words χρoνoς (chronos, meaning time) and $\sigma \overset{´}{\upsilon }\gamma$ (syn, meaning common), which together translate to “sharing common time” or “occurring at the same time” (Pikovsky et al., 2001), a temporal alignment of two systems. Humans have a remarkable tendency to align their physiology and behaviors with the emotional states of others while observing or interacting with them (Chartrand and van Baaren, 2009; Stephens et al., 2010; Ebisch et al., 2012; Tomatsu and Isoda, 2023). This ability to synchronize with others is fundamental to us as social beings (Cross, 2005). Synchrony refers to a relationship that involves at least two units of analysis, whether between two modalities within an individual or between two individuals, which is known as interpersonal synchrony (Zilcha-Mano, 2025). There has been a growing interest in understanding interpersonal synchrony, in terms of physiology; the dynamic coordination of physiological processes, such as heart rate (HR), respiration, electrodermal activity and brain activities between individuals engaged in meaningful interaction (Feldman, 2012; Palumbo et al., 2017). Synchrony in HR between parent and child is thought to be a co-created and mutually adaptive process (Feldman et al., 2011). It is believed to be most prominent during active interactions (Suveg et al., 2016) and HR synchrony was also associated with the mother-child dyad's ability to recover from a negative experience (Woltering et al., 2015). Even before a child is born, HR synchrony has been found between the mother and fetus, in both directions of influence (Nichting et al., 2023). This may explain the connection between fetal HR fluctuations and the mother's physiological and psychological state, as well as how pregnant mothers can perceive certain changes in their unborn child (Ivanov et al., 2009; Van Leeuwen et al., 2009). Researchers also proposed that the HR synchrony may reflect emotional contagion (Hatfield et al., 1993; Lin et al., 2024) and joint experience (Gordon et al., 2020), as observed in studies with couples in romantic relationships across various task settings (Helm et al., 2012; McAssey et al., 2013; Wilson et al., 2018; Coutinho et al., 2021). HR synchrony had also been observed in larger group studies. Such as between performers of a fire walking ritual and the spectators who were related to them, but not observed in unrelated spectators (Xygalatas et al., 2011), and another study showed HR synchrony among concert-goers (Tschacher et al., 2024). These studies highlighted the associations between HR synchrony and empathetic response (Xygalatas et al., 2011) and affectivity and personality (Tschacher et al., 2024).

Traditional self-report measures have generated vast knowledge in therapeutic settings but with the advance of computational technology in regard to the handling of vast amounts of data and the complexity of the analysis, researchers have gained the possibilities to look into other aspects which might be involved in therapeutic change, such as physiology (Zilcha-Mano and Ramseyer, 2020). Coleman, Greenblatt, and Solomon (Coleman et al., 1956) were likely the first to report significant correlations between the HR of therapist and patient during psychoanalytic interviews in 1956 (Tschacher and Meier, 2020), and they suggested that HR synchrony was associated with a greater focus on the client (Palumbo et al., 2017). Similarly, (Kodama et al. 2018) found in their study that HR synchrony occurred during clinically important scenarios and was higher when the therapist showed empathic and attentive behavior. Subsequently, (Tschacher and Meier 2020) demonstrated that dyadic therapeutic HR synchrony was beyond random in their study in a clinical setting and suggested that HR synchrony was associated with therapist's progress and alliance rating (Tschacher and Meier, 2020).

Despite its potential to uncover the physiological mechanisms underlying therapeutic relationships, HR synchrony remains largely underexplored in music therapy research. In a non-clinical setting with healthy individuals and a music therapist, (Neugebauer and Aldridge 1998) recorded the heart rates of 11 dyads during improvisation. The music therapist selected key moments based on musical interactions, and a physiologist then analyzed the plotted HR data graphically and compared it to those key moments to identify significant patterns. The authors concluded that HR changes were linked to relational and communication processes, with greater synchrony during music activities compared to talking or resting. Neugebauer and Aldridge also suggested that the MT intervention created a relational phenomenon that involved both musical and physiological contact between the participants (Neugebauer and Aldridge, 1998). Likewise, (Gordon et al. 2020) found in their non-clinical drumming study that HR synchrony increased significantly during drumming than during the resting state and also suggested that HR synchrony could be the result of shared states and joint participation. A deeper understanding of HR synchrony in MT could provide valuable insights into how therapeutic relationships may manifest at the physiological level. Knowledge of this bio-marker may enhance our comprehension of the mechanism of change and contribute to the advancement in clinical practice. To our best knowledge, no systematic study has been conducted to examine the emergence of HR synchrony between therapist-patient dyads in music therapy and its possible implications. This exploratory case-series study aims to take the first steps in addressing the current gap in music therapy research.

In our previous paper, we compared the nonverbal (NV) synchrony, which we defined as movement coordination between 11 therapist-patient dyads, using a segment size of 10 seconds and a lag of ± 2 s, during unstructured conversations before and after MT interventions and observed a significant increase (*W* = 5, *p* = 0.010, *r* = −0.85). In this paper, we shift our attention to the HR synchrony of these 11 dyads, which we define as the coordinated heart-rate patterns between therapist and patient, while they engage in MT interventions. In accordance with previous studies on motion synchrony (overview in Ramseyer, 2020), we employed windowed cross-correlations of the paired time series to calculate HR synchrony (e.g., Coutinho et al., 2021). Our goal was to understand if and how HR synchrony emerges during MT interventions. We hypothesized that the dyadic HR synchrony emerges beyond chance during MT interventions. Our first research question is:

1. Is the dyadic synchrony observed during the MT interventions significantly greater than the pseudo HR synchrony generated through between-subjects shuffling?

We also aimed to determine whether dyadic HR synchrony during a moment of interest (MOI, see Section 2.2) selected by the music therapist is higher than the average HR synchrony throughout the MT intervention. Our second research question is:

2. Is dyadic HR synchrony higher in MOI segments?

Additionally, we wanted to explore other factors which might be associated with the dyadic HR synchrony that emerged during the MT Interventions, leading us to ask:

3. Is the duration of the MT interventions associated with dyadic HR synchrony?
4. Is the dyadic HR synchrony linked to patients' therapy readiness, as assessed by the patients and the therapist?
5. Is the nonverbal synchrony before and after the MT intervention associated with dyadic HR synchrony?
6. Is the dyadic HR synchrony driven more by patients or the therapist?

To answer question 2, we compared the HR synchrony values of the MOI segments to the average HR synchrony value of that dyad. In addition, the four highest (SYN) and the four lowest (NSYN) HR synchrony segments were highlighted and compared with the MOI segments to examine the temporary overlapping of these events. For questions 3 to 6, we considered factors such as the duration of the MT intervention, the NV synchrony before and after the MT intervention, and the leading characteristics of the HR synchrony. Due to the limited nature of the available dataset and the unorthodox double role of the first author as both the music therapist conducting the MT sessions and the researcher, no general findings were expected.

## 2 Conceptual background

### 2.1 The heart

Roughly the size of a clenched fist, the heart is the primary organ of the circulatory system that pumps blood throughout the body, with the rate and rhythm of the heart's beating depending on its electrophysiology and electrical conduction system (Gray et al., 2005; Schneck, 2015). A heartbeat is primarily initiated by the S-A node, also referred to as the pacemaker, generating action potentials that cause the heart muscles to contract in an orchestrated fashion (Appelhans and Luecken, 2006). The activity of the S-A node is affected by the sympathetic and parasympathetic branches of the autonomic nervous system thereby influencing the heart rate (Appelhans and Luecken, 2006). The electrical activity of the heart can be described by the waveform markings on an electrocardiogram (ECG) which has 3 main components: P wave, QRS complex and the T wave. A ventricular cardiac cycle, or a heartbeat, is represented by the R-R interval, i.e., the distance between two successive R-peaks (Sörnmo and Laguna, 2005). The number of times a heart beats is conventionally measured in beats per minute (BPM) (Andreassi, 2010; King and Lowery, 2023).

HR responds to physical and mental demands bi-directionally and reflects the psychophysical state of an individual (Gramann and Schandry, 2009). HR has been frequently used as a key physiological measure to indicate cardiovascular events in psychophysiology studies (Gramann and Schandry, 2009; Hodges, 2011) owing to the relatively easy and non-invasive method of collecting HR data. HR decreases during relaxation, orientation and attention processes, whereas an increase in HR could be due to factors such as pain and fear stimuli (Gramann and Schandry, 2009), cognitive elaboration or deeper internal focus and less focus on the external environment (Papillo and Shapiro, 1990; Andreassi, 2010).

Short-term fluctuations in HR are almost exclusively stimulus-related, lasting between 1 to 15 s, and are called phasic HR changes. In the classical cognitive approach, an orienting reflex, where attention and perception are enhanced, is when HR demonstrates rapid, brief deceleration to moderate or novel stimulation. From the onset of the stimulus, this reaction has a latency of about 0.5 to 2 s and would have reached its lowest within 2 to 7 s (Vila et al., 2007; Gramann and Schandry, 2009). However, when the stimulus is more emotional, this deceleration takes a longer time (Bradley et al., 2012). A defense response, on the other hand, in which HR accelerates and is associated with reduced perception, occurs when the stimulus is intense or aversive (Vila et al., 2007). The onset of this reaction begins 0.5 to 3 s after the stimulus and typically reaches its highest within 5 and 10 s (Gramann and Schandry, 2009). The maximum deceleration or acceleration can occur between 5 and 15 s after the stimulus, and for real-time analysis in seconds, BPM is generally preferred (Gramann and Schandry, 2009).

The activation of the parasympathetic nervous system lessens with excitable music (Iwanaga et al., 2005) similarly, a higher HR is associated with exciting music, which evokes emotional arousal (Koelsch and Jäncke, 2015). Research has shown that 5 min of exposure to Pachelbel's Canon in D Major is associated with a reduction in sympathetic regulation, whilst 5 min of exposure to intense rhythm heavy metal music from Gamma Ray (Heavy Metal Universe) brought about a reduction of the heart's sympathetic and parasympathetic modulation (da Silva et al., 2014).

### 2.2 Moment of interest

Depending on the context, the population, and the paradigms, various terms have been used to describe powerful and meaningful experiences that encompass a pivotal function that provokes change (Gavrielidou and Odell-Miller, 2017; Coomans, 2018). In this paper, Moment of Interest (MOI) is defined as

“… selected target sequences in a time series of clinically relevant events that are recognized and selected based on personal and/or professional preferences and interests” (Fachner et al., 2019, p. 4).

The music therapist identified MOI segments in which significant changes or new thoughts emerged, crucial for the therapeutic relationship and the patient's progress. This selection was made before calculating the HR synchrony to avoid bias. HR synchrony could provide further insight into this phenomenon, and the measuring instrument should allow for research in clinical practice (Fachner, 2014). More details on MOI in Section 3.4.

## 3 Methods

### 3.1 Sample

The convenient sample consists of 11 patients and one music therapist. All patients in this nested study were recruited based on the inclusion and exclusion criteria of the Right Period Project. To be included in the study, the patients must be admitted as an in-patient of the neurorehabilitation department of the hospital with a principal neurological diagnosis, they must be between the ages of 18 and 99, be able to verbalize and to give written informed consent. Exclusion criteria included pre-existing or newly developed atrial fibrillation or flutter, implanted pacemakers or defibrillators, allergic reactions to the electrodes' adhesive, significant neuropsychological impairments, and medical crises during the study. None of the patients had physical impairments, like hemiplegia, that would affect their movements. Of the 11 patients, seven were females (63.6%) and four were males (36.4%) with ages ranging between 39 and 60 (mean age 51 years, SD = 6.48). One patient was diagnosed with cervical disc disorder with myelopathy, six with lumbar and other intervertebral disc disorders with radiculopathy, one with lumbago with sciatica, one with low back pain, one with cerebral infarction (unspecified) and one with cerebral infarction due to unspecified occlusion or stenosis of cerebral arteries. The music therapist (also first author) who conducted the MT session was female, 43 years old.

### 3.2 Music therapy settings

MT was not a standard treatment at the facility, but all 11 patients received it during their hospital stay. During the 4-day data-collection period of the larger project, each patient received two MT sessions. One of these two sessions was selected and analyzed, based on these criteria: availability of a video of the complete session, which was suitable for movement synchrony analysis, availability of the complete heart-rate data from the patient and the music therapist and the number of segments of MOI, as this study aims to explore the relationship between MOIs and HR synchrony. Prior to the data-collection period, in the process of recruitment, the patients had the opportunity to experience a “get-to-know” MT session with the music therapist where no data was collected. The selected sessions for analysis were therefore all within the first three MT sessions. After the 4-day period, MT session continued in some cases according to patient's therapeutic process, without data collection, until the patients were discharged.

At the start of the session, the patient and the music therapist independently and subjectively rated the patient's level of therapy readiness on separate visual analogue scales (VAS). Therapy readiness can be expressed as

**Figure 1:**
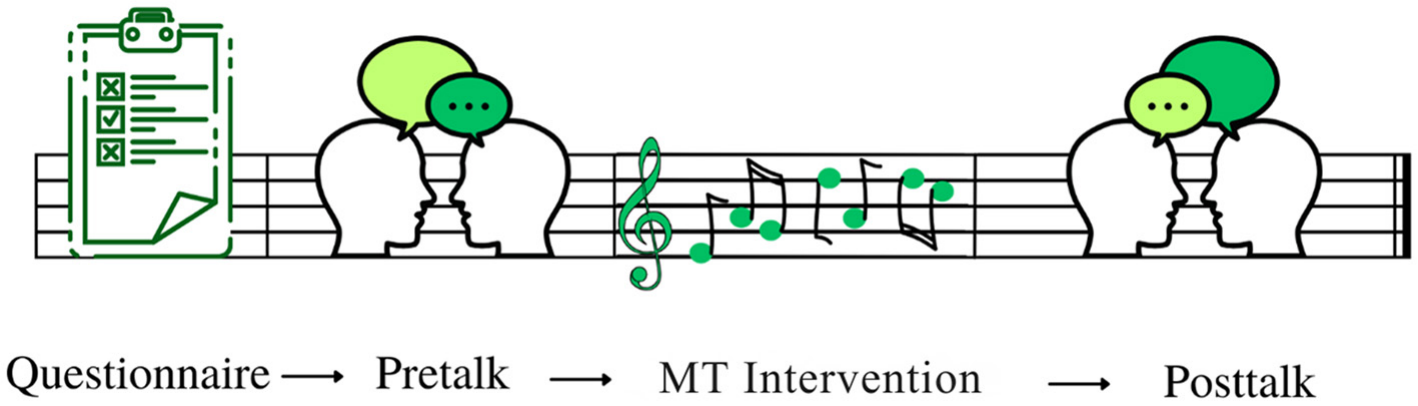
Music therapy process.

“patient's positive attitude and preparedness to enter into a therapeutic relationship for the purpose of resolving problems” (Ogrodniczuk et al., 2009, p. 427),

and to have therapy readiness means that one is motivated to engage in therapy (Rapp et al., 2007). This information remained confidential from the other. The music therapist then initiated an unstructured conversation to assess the patient's mood and momentary condition, allowing them to decide on the session's direction and goals. This was followed by an MT intervention where the type of intervention was tailored to the patient's needs, such as receptive music listening, instrument exploration, improvisation, active music-making, singing, songwriting, and mindfulness exercises with musical elements. It was intended in the study to keep to an authentic and organic condition of actual MT clinical practice; the type of MT interventions was focused on the needs of the patients (reflective clinical practice); thus, the duration of the MT interventions and the unstructured talks were also left unrestricted. Figure 1 shows the process of the MT session in this study. Table 1 shows the type, duration of the MT intervention and MOI segments.

**Table 1:** Type of MT intervention, duration of MT intervention, and MOI segments.

Dyad	Type of MT interventions	MT intervention duration (minutes)	MOI 1 duration (seconds)	MOI 2 duration (seconds)	MOI 3 duration (seconds)	MOI 4 duration (seconds)	MOI 5 duration (seconds)	Total MOI duration (seconds)	Average MOI duration (seconds)
P018	•Drumming •Listening to patient's preferred music	29.75	32	92	92	152	NA	368	92
P019	•Mindfulness exercise: relaxation, here/now •Songwriting/singing original song	29.13	122	92	92	122	NA	428	107
P025	•Mindfulness exercise: breathing •Voice work, Singing together	26.05	92	92	92	92	62	430	86
P028	•Mindfulness exercise: deep listening •Improvisation: instruments •Music association/discussion	23.98	62	32	62	62	32	250	50
P031	•Improvisation: instruments •Drumming	35.02	32	32	92	122	152	430	86
P032	•Improvisation: only voice •Music association/discussion	11.98	62	62	32	62	NA	218	54.5
P035	•Instrument exploration •Improvisation: instruments, instruments and voice	20.15	62	92	62	32	92	340	68
P037	•Instrument exploration •Improvisation: instruments	15.12	62	62	132	62	NA	318	79.5
P047	•Mindfulness exercise: deep listening •Voice work •Improvisation: instruments	34.53	62	62	32	32	62	250	50
P048	•Instrument exploration •Improvisation: instruments, instruments and voice	27.87	62	182	122	92	62	520	104
P057	•Listening to patient's preferred music •Singing together •Music association/discussion	28.23	62	62	62	62	NA	248	62

### 3.3 Video data collected

The sessions were recorded digitally with an iPhone 8 camera on a fixed stand, about two and a half meters away from the middle of the dyad. It captured 30 frames per second without autofocus and did not obstruct any “natural” activity of the MT session in order to reduce demands on the patients (Heath et al., 2010). As a digital medium, video materials were used for quantitative analysis, using the Motion Energy Analysis programme (MEA; Ramseyer, 2020; Yap et al., 2022), the selection of MOI, and qualitative content analysis of the therapy interactions.

### 3.4 Selection of moment of interest

Each session was summarized using the video recordings and notes written by the music therapist immediately after the session, and the music therapist selected four or five MOI segments from each MT intervention. The selection of MOI segments was performed before the calculation of HR synchrony to avoid bias. As usual in clinical practice, the MOI segments were intentionally selected for their therapeutic significance and transformational potential from the music therapist's perspective (Wosch and Wigram, 2007; Fachner et al., 2019). The justification of each MOI segment was documented (Yap, 2024). The main focus was on the relational connection; emergence of patient's musical-self; patient's emotional expression and processing; patient's insight and awareness; patient's cultural and personal identity. Below are some examples of reasons for selection.

P018 MOI 4 (Focus-Relational Connection, Cultural Identity, Insight):

“*The patient wanted to share a song of worship that he particularly liked. At first, he found the instrumental version, but since he had mentioned the words were important to him, the music therapist asked to listen to the sung version. The music therapist saw this segment as an intimate moment, where the patient revealed something very precious to him, and something that had ‘held things up for his life,' as he would later explain in the posttalk.”*

P019 MOI 4 (Focus-Empowerment, Emotional Regulation):

“*The music therapist felt that this segment demonstrated how the patient was supported by her own personal song to regulate her emotions and achieve a more driven and positively oriented outcome. Furthermore, it demonstrated that the patient was empowered to choose this song as a resource at home.”*

P037 MOI 3 (Focus-Empowerment, Emotional Expression):

“*The patient played with both hands. Not simultaneously, but more like a conversation between her right and left sides. She was very engrossed and determined to stay on it despite the music therapist's musical proposal to change. The music therapist gave in and supported her in this inner dialogue. With that, they were able to “play” together. The patient demonstrated her independence and gave the impression that the inner dialogue she was having was important enough to demand space for it. This was a moment of discordance, but it was nevertheless something positive.”*

P048 MOI 4 (Focus-Emotional Processing, Insight, Acceptance):

“*The music therapist asked the patient what he needed. The patient said that he needed affection, love, and understanding. These words appeared to be difficult for the patient to say out loud. He had to breathe deeply between the words and looked to the music therapist for reassurance. After a long pause, he said, “I need help.” Then he couldn't go on. It was a moment of realisation, of acceptance.”*

These MOI segments were considered clinically relevant events and crucial for the therapeutic relationship and the patient's progress. Although such moments varied in duration, they were captured in segments of multiples of 30 s plus 2 s, to align with the HR synchrony segment duration, with one exception, where it was not possible (see Table 1).

### 3.5 Heart-rate data

During the MT intervention, HR data were recorded on a single-channel electro-cardiogram sensor eMotion produced by Mega Electronic in Finland. The ECG sensor sampled at 1,000 Hz with an accuracy of 1 ms (Remes, 2015) and recorded the time difference between consecutive heartbeats; the R-R interval. The ECG sensor has been declared by its manufacturer to be in conformity with the provisions of the Council Directive 93/42/EEC (and related to Finnish national laws) and the amended Directive 2007/47/EC concerning medical devices. The raw R-R data consists of a time series made up of successive RR intervals in milliseconds and was stored in the Lebensfeuer^®^ Analyse portal (Autonom Health, 2021). The same ECG sensors and portal have been used in other studies (Laczika et al., 2013; Janssen et al., 2020).

Unlike a standard time series, where the data points are collected at fixed intervals, the raw R-R data is a “quasi time series.” It is a sequence of data that was taken over time, but the data points are not fixed or at regular intervals. Therefore, the different time series would not have a common base and these cannot be used to compare between subjects (Ferrer and Helm, 2013). The raw R-R value was therefore transformed into a discrete time series through linear interpolation into milliseconds per second. Filtering procedure was also implemented to correct for artifacts by the Autonom Health platform (Autonom Health, 2021) following the guidelines set in 1996 by the Task Force of the European Society of Cardiology the North American Society of Pacing Electrophysiology (1996). The order of filtering in this study was as follows:

- “End Detection Filter”: All values greater than 2,000 ms are reset to 1,300 ms
- “Movement Filter”: Windows are formed from 60 values. All values that deviate more than 25 % from the median of this window are reset to 25 % deviation
- “Trend Filter”: Linear regression is calculated from a window of 1,500 data points and the data series are subtracted from this. This eliminates a trend
- “Rapid Change Filter”: Changes between two consecutive values, which account for more than 35% are reset
- “Threshold Filter”: Values that are above or below the upper and lower limits of 300 ms and 2,000 ms are reset to these limits.
- The result of this step is saved under “timeconsistent_filtered”

A visual inspection of the raw unfiltered R-R data and the time consistent filtered R-R data, of the period of analysis (Min duration = 11.98 min, Max duration = 35.92 min) was undertaken to check the filtering procedure corrected artifacts but not masked deviations from normal heartbeat. An example of this inspection is provided (see Appendices 1, 2). Lastly, the filtered time-consistent R-R intervals were converted to beats per minute (BPM) using the formula BPM = 60,000/R-R interval, time-stamped, and the resulting data at 1 Hz were used to calculate HR synchrony.

### 3.6 Calculation of HR synchrony

HR synchrony was analyzed during the MT interventions (*N* = 11). The average MT intervention duration was 25.62 min, with a minimum of 11.98 min, a maximum of 35.02 min and a standard deviation of 7.34 min. Time-consistent HR data were analyzed using the rMEA package for the free software environment RStudio (RStudio Team, 2020; Kleinbub and Ramseyer, 2021). The HR synchrony of 50 selected MOI segments was also analyzed using rMEA.

The rMEA package evaluates cross-correlation within a specific window, i.e., a restricted portion of the original time-series where local stationarity can be assumed, and applies a lag analysis, as the coupling observed in intersubjective contexts is often characterized by a delay between similar features in two time-series. A lag analysis enables a more precise estimation of synchrony and provides information about who is leading, i.e., occurring first (Kleinbub and Ramseyer, 2021). The leading in synchrony may provide important information particularly in contexts where the dyad has different roles such as therapist and patient (Tschacher et al., 2025). Leading may be influenced by the therapy orientation of the therapist (Tschacher et al., 2025) and the patient's pathology (Kupper et al., 2015). As a dyadic metric, Leading/Following characteristics in HR synchrony can occur when changes occur in patients' HR that are then reflected by therapist's HR, or when therapist's HR changes are subsequently mirrored by patient's HR (Asher et al., 2021). Since MT is based on a process of mutual relations, the implications of leading are certainly relevant. Researchers in psychotherapy have suggested that therapist leading in HR synchrony was linked to therapist collecting information and providing advice (Kodama et al., 2018), and in a study of patients with Social Affective Disorder, patient leading was positively associated with low HR synchrony (Asher et al., 2021). The cross-correlation algorithm implemented in rMEA involves 3 phases:

1. Simultaneous (or lag-0) synchrony of the two persons' signals within a window *W* is calculated using simple Pearson correlation
2. Then the cross-correlation is calculated again, by moving one of the two time series forwards and backwards by 1 s steps, until the lag *L* interval is achieved.
3. In the last phase, the window is moved by an increment *I*, either smaller (for overlapping windows) or in our study, equal (consecutive windows) to the window size. The cycle repeats until the whole duration is analyzed.

This algorithm results in a matrix of correlations, with 2*L* + 1 columns, one for each lag value from –*L* to *L*, and (*D – W* + 1)/*I* rows, rounded up, one for each moving window (Kleinbub and Ramseyer, 2021).

Temporal dynamics of HR synchrony are still largely unknown; current literature investigating synchrony on the cardiac level, the applied methods, selection of segment size, and lags is inconsistent (Palumbo et al., 2017; Gordon et al., 2020). HR synchrony in this study was calculated using windowed cross-correlation in absolute value for every 30 s with a lag of ± 2 s (MEAccfall = all_lags). The choice of 30 s segments provided a good balance between minimally necessary data-points available per segment (i.e., *n* = 30), and because it has been a default value in the domain of MEA-based interaction research (e.g., Tschacher et al., 2014; Völter et al., 2024), and other recent studies in the domain of psychotherapeutic and counseling interaction have also used ≥30 s segments (e.g., Hoffmann et al., 2024; Peysachov et al., 2025). This amount of resulting datapoints is also aligned with the recommendations of the rMEA authors of ≥30 values per segment. Considering that the heart's reaction has a latency of about 0.5 to 2 s after the onset of the stimulus (Gramann and Schandry, 2009) and phasic heart rate changes generally concluded within 15 s (Vila et al., 2007) with the maximum deceleration or acceleration between 5 and 15 s after the stimulus (Gramann and Schandry, 2009), the parameters chosen in this study should allow the capture of both the stimulus and the heart's response during the dyadic interaction as the latency of HR reaction happens and concludes in this range. Using rMEA, the leading characteristic of HR synchrony was identified, as well as the average HR synchrony during the MT interventions.

### 3.7 Determining the highest and lowest HR synchrony segments

**Figure 2:**
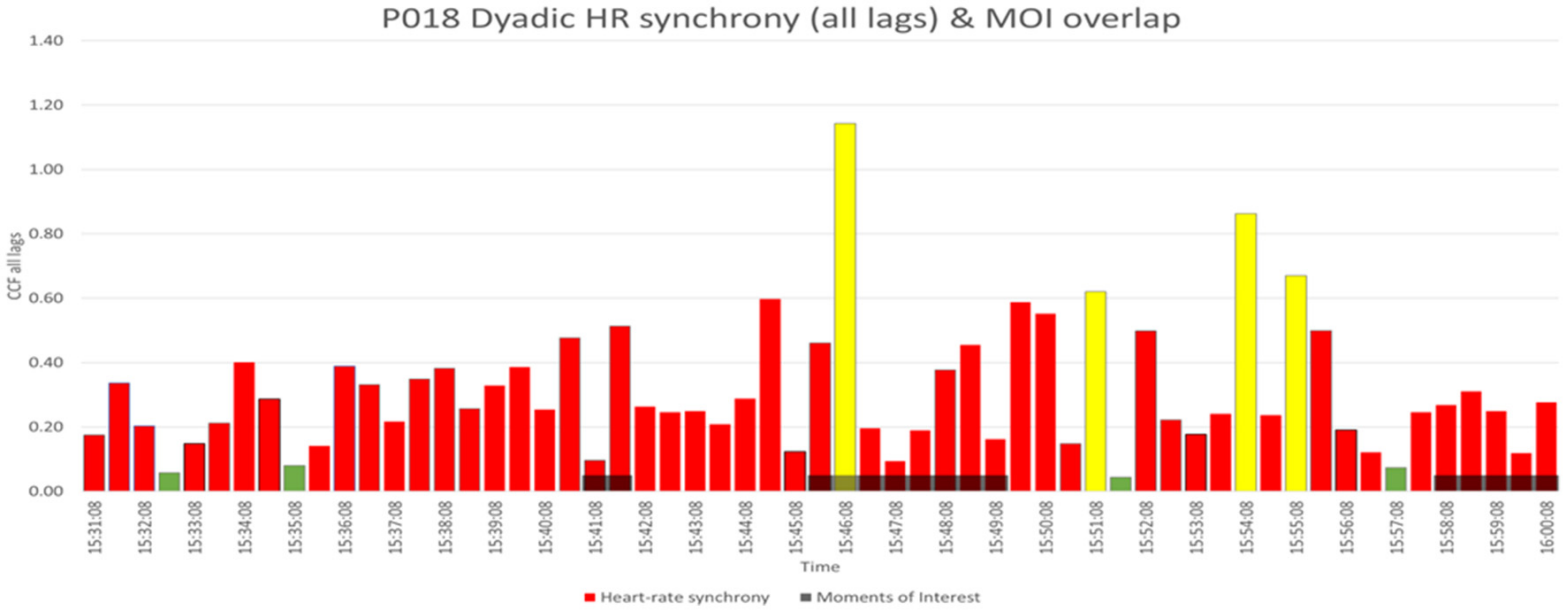
Dyadic heart-rate synchrony in 30 s segments of P018; SYN, NSYN, and MOI segments.

Four SYN segments and four NSYN segments from the entire MT intervention from every dyad were highlighted for the subsequent qualitative video analysis. The HR synchrony of the MOI segments was also analyzed, and the leading characteristics of each segment were found. Figure 2 is an example from a dyad P018, which shows the dyadic HR synchrony in 30 s segments graphically, where the x-axis is the timeline, and the y-axis shows Fisher's Z standardized correlation values. The SYN segments are highlighted in yellow, the NSYN segments are green, and the moments of interest (MOI) are black.

### 3.8 Heart-rate pseudosynchrony

To discriminate whether the synchrony is indeed the result of attunement or is merely the consequence of individual periodic patterns, the actual HR synchrony was compared to the HR pseudosynchrony in rMEA. This HR pseudosynchrony is the result of between-subject shuffling, combining a dyad's time series with a random time series from the entire pool of observations (*N* = 220, i.e., 11 × 10 × 2), resulting in the creation of pseudo-datasets (Ramseyer and Tschacher, 2010; Moulder et al., 2018; Kleinbub and Ramseyer, 2021). If the HR synchrony in the study were genuine, it would exceed the level of pseudosynchrony and would yield a greater effect size (Ramseyer and Tschacher, 2010).

### 3.9 Event occurrence analysis and associations

This study also explored the temporal perspective of HR synchrony. Using simple linear regression in Jamovi 2.6 (The Jamovi Project, 2025), we looked at the association between HR synchrony and intervention duration, and with Microsoft Excel Office 16 (Microsoft Corporation, 2018), we investigated when SYN, NSYN, and MOI segments occurred more frequently, and the amount of overlapping between MOI and the SYN or NSYN segments was also examined. We were also interested to see if there was a particular “sweet spot” for the emergence of dyadic HR synchrony, thus, we grouped HR synchrony values of all 30 s segments into 5 min blocks, which aligns with common hospital therapy duration allocation, with Microsoft Excel Office 16, the mean HR synchrony of these blocks was calculated, the highest HR synchrony value from each block was selected, and the number of top five-percent values were marked.

We explored the relationship between the HR synchrony and Patient/Therapist leading during SYN, NSYN, and MOI segments, using statistical tools such as Wilcoxon, chi-square and Fisher's exact test.

### 3.10 Statistics

Using Jamovi 2.6 (The Jamovi Project, 2025), normality assessment of mean HR synchrony across dyads (*N* = 11) showed that the Shapiro-Wilk test (*W* = 0.938, *p* = 0.498) did not reject the null hypothesis of normality. Given the small sample size, this should be interpreted with caution; however, there is no strong indication of deviation from normality. Accordingly, we proceeded with parametric tests for analyzing dyadic mean values. Table 2 shows the descriptives and distribution of HR synchrony between dyads.

**Table 2:** Descriptives and distribution of HR synchrony between dyads.

HR synchrony	
*N*	11
Mean	0.301
Median	0.298
Standard deviation	0.022
Minimum	0.260
Maximum	0.348
Shapiro-Wilk W	0.938
Shapiro-Wilk p	0.498

When 30 s segments of each dyad were assessed, 10 out of 11 dyads showed statistically significant deviations from normality (*p* < 0.05). However, the Shapiro-Wilk (*W* = 0.76 to 0.93) values suggested a moderate rather than an extreme deviation from normality. Furthermore, most dyads showed a right-skewed distribution (mean > median), with most values clustered on the lower end (hard lower limit at 0) and a longer tail extending toward the higher values. Table 3 shows the descriptives and distribution of HR synchrony within dyads.

**Table 3:** Descriptives and distribution of HR synchrony within dyads.

Dyad	P018	P019	P025	P028	P031	P032	P035	P037	P047	P048	P057
*N*	59	58	52	47	70	23	40	30	69	55	56
Mean	0.310	0.286	0.309	0.297	0.317	0.292	0.292	0.260	0.348	0.298	0.305
Median	0.254	0.249	0.262	0.206	0.283	0.314	0.244	0.208	0.277	0.252	0.232
Standard deviation	0.202	0.161	0.195	0.255	0.210	0.148	0.194	0.133	0.239	0.191	0.244
Minimum	0.044	0.066	0.038	0.053	0.016	0.064	0.066	0.067	0.043	0.072	0.039
Maximum	1.14	0.750	0.951	1.36	1.15	0.581	0.850	0.501	1.02	1.07	1.32
Shapiro-Wilk W	0.872	0.927	0.928	0.777	0.863	0.945	0.898	0.928	0.910	0.877	0.761
Shapiro-Wilk p	< 0.001	0.002	0.004	< 0.001	< 0.001	0.228	0.002	0.043	< 0.001	< 0.001	< 0.001

Therefore, when analyzing HR synchrony across time within dyads, non-parametric statistics were more appropriate. The standard deviations (0.13 to 0.25) indicated moderate variability within dyads and the wide range within dyads suggested heterogeneous synchrony dynamics over time.

The normality assessment of average HR synchrony in MOI segments across dyads (*N* = 11) resulted in a Shapiro-Wilk *W* = 0.801, *p* = 0.010, indicating that the distribution was not normal and highly skewed at 1.38. Therefore, when analyzing HR synchrony on the group level, non-parametric tests were applied. Table 4 shows the HR synchrony descriptives and distribution of MOI segments between dyads.

**Table 4:** MOI HR synchrony descriptives and distribution between dyads.

MOI HR synchrony	Values
*N*	11
Missing	0
Mean	0.323
Median	0.282
Standard deviation	0.093
Minimum	0.220
Maximum	0.507
Skewness	1.380
Std. error skewness	0.661
Shapiro-Wilk W	0.801
Shapiro-Wilk p	0.010

When MOI segments of each dyad were assessed, all but one dyad showed no statistically significant deviation from normality (*W* = 0.81 to 0.94), suggesting that per-dyad MOI segment distributions were approximately normal. Table 5 presents the HR synchrony descriptive statistics and the distribution of MOI segments within dyads. An alpha level of 0.05 was used for all statistical tests.

**Table 5:** MOI HR synchrony descriptives and distribution within dyad.

Dyad	P018	P019	P025	P028	P031	P032	P035	P037	P047	P048	P057
*N*	4	4	5	5	5	4	5	4	5	5	4
Mean	0.358	0.279	0.282	0.490	0.303	0.261	0.262	0.220	0.507	0.308	0.282
Median	0.326	0.299	0.231	0.571	0.250	0.213	0.279	0.211	0.490	0.260	0.331
Standard deviation	0.121	0.060	0.113	0.290	0.168	0.146	0.087	0.045	0.253	0.093	0.107
Minimum	0.256	0.190	0.166	0.149	0.173	0.148	0.140	0.179	0.144	0.209	0.121
Maximum	0.524	0.327	0.44	0.765	0.59	0.472	0.349	0.278	0.855	0.417	0.343
Shapiro-Wilk W	0.902	0.816	0.917	0.854	0.811	0.851	0.932	0.923	0.941	0.856	0.695
Shapiro-Wilk p	0.443	0.135	0.509	0.207	0.100	0.230	0.610	0.556	0.675	0.215	0.010

## 4 Results

### 4.1 HR synchrony vs. pseudo HR synchrony

The first research question asked if the dyadic synchrony observed during the MT interventions is significantly greater than the pseudo HR synchrony generated through between-subjects shuffling. We found that, on the individual level, only one dyad (P037) had an intervention average less than the average pseudo HR synchrony (0.2860); the group average HR synchrony of 11 dyads during MT intervention (0.3012) was more than the average pseudo HR synchrony. Table 6 shows each dyad's average, highest and lowest HR synchrony values during MT intervention and their leading characteristics. The combined averages and the medians are also provided.

**Table 6:** HR synchrony and leading characteristics during MT intervention.

Dyad	HR synchrony intervention average	Therapist lead	Patient lead	Leading	Highest HR synchrony intervention	Lowest HR synchrony intervention
P018	0.3098	0.3172	0.3018	Therapist	1.1423	0.0437
P019	0.2861	0.2729	0.3002	Patient	0.7503	0.0656
P025	0.3089	0.2974	0.3068	Patient	0.9513	0.0376
P028	0.2967	0.2676	0.3218	Patient	1.3628	0.0532
P031	0.3168	0.3252	0.3097	Therapist	1.1503	0.0164
P032	0.2918	0.2944	0.2887	Therapist	0.5809	0.0637
P035	0.2919	0.2706	0.3097	Patient	0.8500	0.0656
P037	0.2604	0.2491	0.2797	Patient	0.5012	0.0671
P047	0.3485	0.3490	0.3415	Therapist	1.0228	0.0428
P048	0.2977	0.2730	0.3194	Patient	1.0701	0.0716
P057	0.3051	0.3108	0.2898	Therapist	1.3203	0.0392
Mean	0.3012	0.2934	0.3063	NA	0.9729	0.0515
Median	0.2977	0.2944	0.3068	NA	1.0228	0.0532
Standard deviation	0.0217	0.0300	0.0174	NA	0.2804	0.0171

A paired sample *t*-test also revealed significant differences between HR synchrony and pseudo HR synchrony (*N* = 220, *p* = 0.047; *d* = 0.48) with a medium effect size. These results indicate that the dyadic HR synchrony found in the data during MT intervention was genuine and the hypothesis that the dyadic HR synchrony occurs at a level significantly greater than chance is supported by the data. Although the *p*-value of 0.047 is very close to non-significance, we decided to adhere to this conventional cut-off value. Cohen's d effect sizes support the strength of the effect, which here ranged at an average effect size (*d* = 0.48).

**Figure 3:**
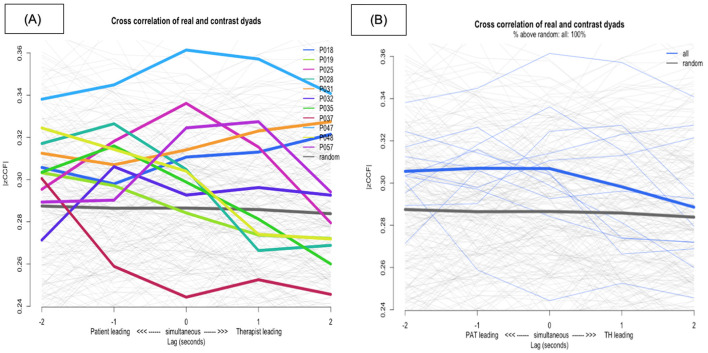
**(A)** Cross-correlation of individual dyad heart-rate synchrony above pseudo heart-rate synchrony. **(B)** Cross-correlation of grouped heart-rate synchrony above pseudo heart-rate synchrony or lag-plot of grouped heart-rate synchrony.

Figure 3A shows the plot of individual dyad HR synchrony (all lags) against the pseudo HR synchrony in gray. Figure 3B presents the lag plot of grouped HR synchrony (all lags) against the pseudo HR synchrony. For both Figures 3A, B, the y-axis |zCCF| is Fisher's Z absolute mean cross-correlation and the x-axis represents the lags, where left of zero (negative values) indicates patient leading and right of zero (positive values) indicates therapist leading.

### 4.2 HR synchrony during MOI

The second question asked whether dyadic HR synchrony in Moment of Interest (MOI) segments is higher than the average HR synchrony across MT interventions. Comparing the average HR synchrony of MOI segments to their corresponding intervention HR synchrony, only four cases were higher (Table 7). The grand average of all MOI segments (0.3229) was higher than that of the grand intervention average (0.3012), but Wilcoxon one sample *t*-test indicated that the difference was not significant (*W* = 32.0, *p* = 0.966, *r* = −0.0303). Comparing HR synchrony of all the MOI segments (Table 8) to their respective intervention average revealed that 24 (48%) of the segments had higher HR synchrony measurements than the corresponding intervention's average, and 26 (52%) of them had lower HR synchrony measurements than the corresponding intervention's average.

**Table 7:** HR synchrony and characteristics during MOI segments vs. during MT intervention.

Dyad	Average HR synchrony during MOI	Average HR synchrony during intervention	MOI higher	Highest HR synchrony during intervention	Lowest HR synchrony during intervention
P018	0.3579	0.3098	YES	1.1423	0.0437
P019	0.2788	0.2861	NO	0.7503	0.0656
P025	0.2825	0.3089	NO	0.9513	0.0376
P028	0.4896	0.2967	YES	1.3628	0.0532
P031	0.3028	0.3168	NO	1.1503	0.0164
P032	0.2613	0.2918	NO	0.5809	0.0637
P035	0.2623	0.2919	NO	0.8500	0.0656
P037	0.2199	0.2604	NO	0.5012	0.0671
P047	0.5073	0.3485	YES	1.0228	0.0428
P048	0.3083	0.2977	YES	1.0701	0.0716
P057	0.2816	0.3051	NO	1.3203	0.0392
Mean	0.3229	0.3012	YES	0.9729	0.0515
Median	0.2825	0.2977	YES	1.0228	0.0532
Standard deviation	0.0933	0.0217	YES	0.2804	0.0171

**Table 8:** Leading characteristics and HR synchrony of all MOI segments, and comparison to the average HR synchrony of dyad's MT intervention.

Dyad	MOI	HR synchrony MOI	HR synchrony intervention average	Leading	HR synchrony MOI > HR synchrony intervention average	Percent of yes	Percent of no
P018	MOI 1	0.3690	0.3098	Patient	YES		
P018	MOI 2	0.5236	0.3098	Therapist	YES		
P018	MOI 3	0.2832	0.3098	Therapist	NO		
P018	MOI 4	0.2558	0.3098	Therapist	NO	50%	50%
P019	MOI 1	0.3266	0.2861	Patient	YES		
P019	MOI 2	0.1904	0.2861	Therapist	NO		
P019	MOI 3	0.2964	0.2861	Patient	YES		
P019	MOI 4	0.3018	0.2861	Patient	YES	75%	25%
P025	MOI 1	0.4403	0.3089	Therapist	YES		
P025	MOI 2	0.2184	0.3089	Therapist	NO		
P025	MOI 3	0.3570	0.3089	Patient	YES		
P025	MOI 4	0.1659	0.3089	Therapist	NO		
P025	MOI 5	0.2309	0.3089	Patient	NO	40%	60%
P028	MOI 1	0.5709	0.2967	Patient	YES		
P028	MOI 2	0.2186	0.2967	Therapist	NO		
P028	MOI 3	0.7450	0.2967	Patient	YES		
P028	MOI 4	0.1487	0.2967	Patient	NO		
P028	MOI 5	0.7650	0.2967	Therapist	YES	60%	40%
P031	MOI 1	0.5897	0.3168	Patient	YES		
P031	MOI 2	0.3040	0.3168	Patient	NO		
P031	MOI 3	0.1978	0.3168	Therapist	NO		
P031	MOI 4	0.1726	0.3168	Therapist	NO		
P031	MOI 5	0.2500	0.3168	Patient	NO	20%	80%
P032	MOI 1	0.1796	0.2918	Therapist	NO		
P032	MOI 2	0.4721	0.2918	Therapist	YES		
P032	MOI 3	0.1478	0.2918	Therapist	NO		
P032	MOI 4	0.2455	0.2918	Patient	NO	25%	75%
P035	MOI 1	0.2786	0.2919	Therapist	NO		
P035	MOI 2	0.3336	0.2919	Patient	YES		
P035	MOI 3	0.2111	0.2919	Therapist	NO		
P035	MOI 4	0.3489	0.2919	Patient	YES		
P035	MOI 5	0.1395	0.2919	Therapist	NO	40%	60%
P037	MOI 1	0.2783	0.2604	Therapist	YES		
P037	MOI 2	0.2323	0.2604	Therapist	NO		
P037	MOI 3	0.1791	0.2604	Patient	NO		
P037	MOI 4	0.1899	0.2604	Patient	NO	25%	75%
P047	MOI 1	0.4898	0.3485	Patient	YES		
P047	MOI 2	0.4838	0.3485	Therapist	YES		
P047	MOI 3	0.8553	0.3485	Therapist	YES		
P047	MOI 4	0.5630	0.3485	Therapist	YES		
P047	MOI 5	0.1445	0.3485	Patient	NO	80%	20%
P048	MOI 1	0.2573	0.2977	Patient	NO		
P048	MOI 2	0.2604	0.2977	Therapist	NO		
P048	MOI 3	0.4166	0.2977	Patient	YES		
P048	MOI 4	0.3977	0.2977	Patient	YES		
P048	MOI 5	0.2093	0.2977	Therapist	NO	40%	60%
P057	MOI 1	0.3234	0.3051	Patient	YES		
P057	MOI 2	0.3428	0.3051	Therapist	YES		
P057	MOI 3	0.3392	0.3051	Therapist	YES		
P057	MOI 4	0.1209	0.3051	Therapist	NO	75%	25%
Results					YES 24 NO 26	48%	52%

**Figure 4:**
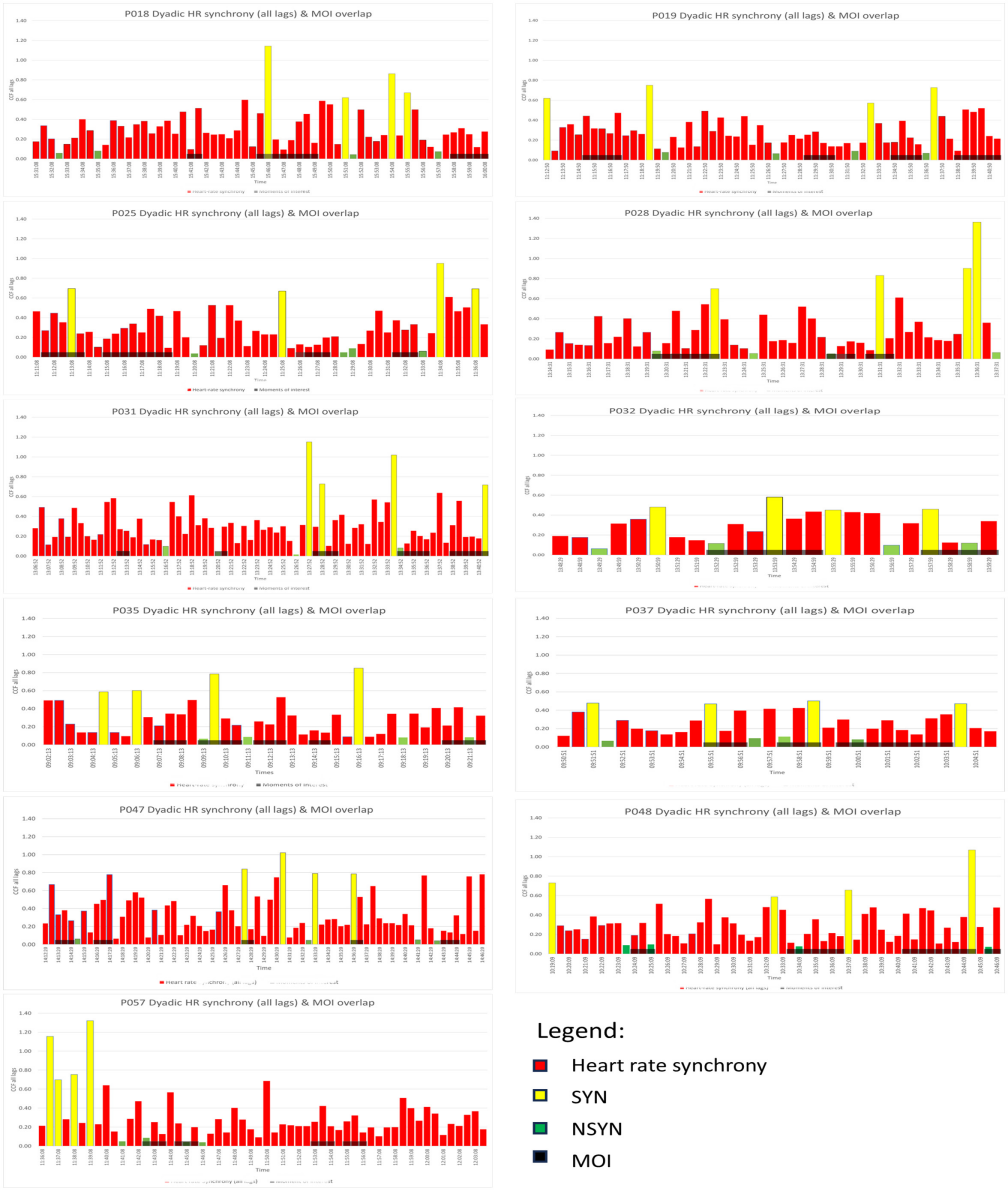
Distribution of the four highest HR synchrony (SYN), the four lowest (NSYN) and the therapist-selected Moment of Interest (MOI) segments.

There are altogether 44 segments of SYN segments, 44 segments of NSYN segments and 50 MOI segments. Figure 4 shows the HR synchrony and the distribution of SYN, NSYN and MOI in 30 s segments. Table 9 shows that whilst NSYN segments were equally distributed between the first and the second halves of the respective MT intervention, SYN and MOI segments occurred more often in the second half. Sixteen SYN segments took place in the first half, 26 occurred in the second half of the MT interventions, and 2 SYN segments were between the two halves. Twenty MOI segments took place in the first half, 27 in the second half and 3 in between. More of these segments were in the second half than in the first half, with a ratio of 74:57.

**Table 9:** Summary of SYN, NSYN, and MOI segments occurrence.

Segments	First half	Second half	In between
SYN	16	26	2
NSYN	21	21	2
MOI	20	27	3
TOTAL	57	74	7

3800 s of the MT interventions were selected as MOI segments (Table 10); 589 s of MOI segments (15.50%) overlapped with either SYN or NSYN segments; and 11 MOI segments overlapped with SYN segments, 13 with NSYN segments, and three overlapped with both. In terms of duration, more MOI segments overlapped with SYN segments (316 s, 53.65%) than with NSYN segments (273 s, 46.35%).

**Table 10:** Summary of MOI overlaps.

MOI overlaps	Frequency/ Duration/ Percentage
Total MOI segments overlap with SYN only	11
Total MOI segments overlap with NSYN only	13
Total MOI segments overlap with SYN & NSYN	3
Total MOI overlap with SYN	316 s
Total MOI overlap with NSYN	273 s
Total MOI overlap	589 s
Total MOI overlap to total MOI (%)	15.50
Total MOI overlap with SYN to total MOI overlap (%)	53.65
Total MOI overlap with SYN to total MOI (%)	8.32
Total MOI overlap with NSYN to MOI overlap (%)	46.35
Total MOI overlap with NSYN to total MOI (%)	7.18

### 4.3 Association between intervention duration and HR synchrony

**Figure 5:**
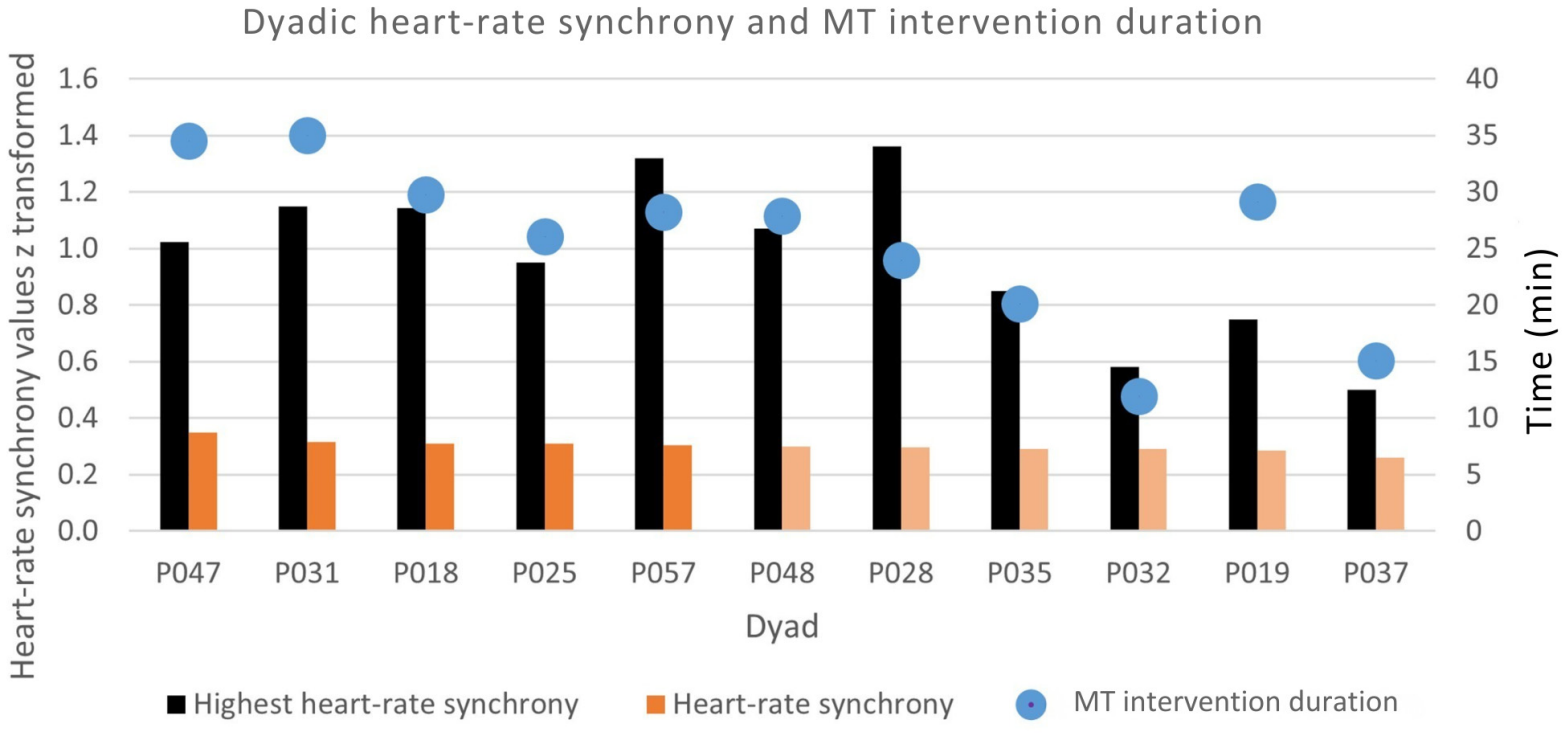
Heart-rate synchrony and music therapy intervention duration.

Our results indicated that HR synchrony is significantly positively associated with MT intervention duration [*F*_(1, 9)_ = 10.5, *p* = 0.010], with an *R*^2^ = 0.539. In addition, MT intervention duration was positively correlated to the highest HR synchrony value of each dyad [*F*_(1, 9)_ = 6.78, *p* = 0.029], with an *R*^2^ = 0.430, and negatively correlated to the lowest HR synchrony value of each dyad [*F*_(1, 9)_ = 6.04, *p* = 0.036], with an *R*^2^ = 0.402. The average intervention HR synchrony is 0.301, and the average highest HR synchrony is 0.973 (Table 4). Figure 5 illustrates that HR synchrony during the MT intervention of dyads P047, P031, P018, P025, and P057, marked as darker orange bars, was higher than the mean of 0.301. The MT intervention duration represented by the blue dots indicated that MT interventions of these dyads also lasted more than 25 min. The black bars indicate the highest HR synchrony value for each dyad.

Grouping all HR synchrony segments into blocks of 5 min, the results showed that the highest HR synchrony value occurred in the 20–25-min block, which also had the greatest mean HR synchrony compared to other blocks and had the most number of top five-percent HR synchrony values (Table 11).

**Table 11:** HR synchrony in 5 min blocks.

Time (min)	Mean HR synchrony block	Highest HR synchrony block	Number of top 5% segment
0–5 min	0.3066 ± 0.2066	1.320328	6
5–10 min	0.3046 ± 0.1724	0.786141	4
10–15 min	0.2801 ± 0.1706	0.849982	1
15–20 min	0.2825 ± 0.2063	1.14226	5
20–25 min	**0.3529** ± 0.2618	**1.362776**	**9**
25–30 min	0.3223 ± 0.2225	1.070093	4
30–35 min	0.3142 ± 0.2428	0.780097	3

The highest values are formated in bold.

### 4.4 Association between therapy readiness and HR synchrony

No association was found between the patient's self-reported therapy readiness [VAS (P)] and dyadic HR synchrony. However, a significant negative correlation was detected between heart-rate synchrony and the therapist's assessment of the patient's therapy readiness [VAS(T)] [*F*_(1, 9)_ = 5.82, *p* = 0.039], with an *R*^2^ = 0.393.

### 4.5 Association between nonverbal synchrony and HR synchrony

We also found no direct association between HR synchrony and the NV synchrony which we have reported in our last paper (Yap et al., 2022).

### 4.6 Association between leading characteristics and HR synchrony

Comparing therapist leading and pseudo therapist leading in HR synchrony during MT intervention showed no significant difference (*N* = 220, *p* = 0.380; *d* = 0.23). On the other hand, a significant difference was revealed between patient leading and pseudo patient leading (*N* = 220, *p* = 0.005; *d* = 0.52) with a medium effect size.

Correlation matrix showed that mean intervention HR synchrony was correlated significantly to HR synchrony led by patient (PLead) and HR synchrony led by therapist (TLead). We found no significant difference between TLead (*M* = 0.29 ± 0.03) and PLead (*M* = 0.31 ± 0.02) conditions; *t*_(10)_ = −1.56, *p* = 0.151.

**Figure 6:**
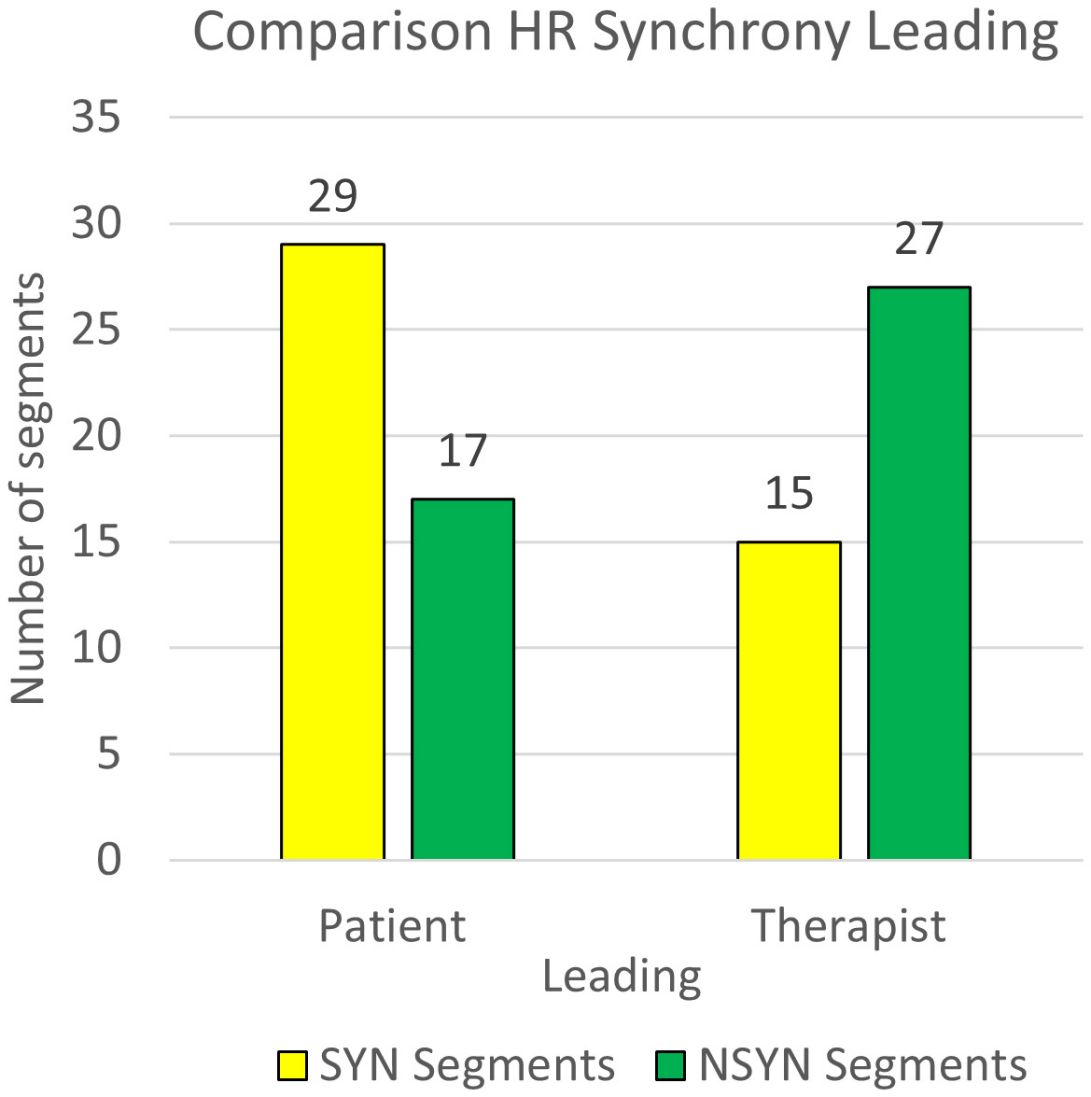
Plot of heart-rate synchrony leading in SYN and NSYN segments.

However, we observed that HR synchrony seemed to be predominant led by patient during the highest SYN segment of each dyad (7:4) and in contrast, TLead was prevalent during the lowest NSYN segments (7:4; Table 12). To explore if there was a pattern in HR synchrony leading during SYN and NSYN segments, we conducted two complementary analyses. First, a contingency table analysis examined the association between HR synchrony leading (PLead vs. TLead) and Segment Type (SYN vs. NSYN) for all 11 dyads (Table 13; Figure 6). A chi-square test revealed a significant association between HR synchrony leading and segment type, χ^2^(1) = 6.56, *p* = 0.010, which was further supported by Fisher's exact test, *p* = 0.018 (Table 14). HR synchrony during SYN segments was more frequently led by patients, while HR synchrony during NSYN segments were more commonly led by therapists. Second, a paired-samples analysis compared, within each dyad (*N* = 11), the number of patient leading in SYN segments (*M* = 2.64 ± 1.03) to patient leading in NSYN segments (*M* = 1.55 ± 0.69). The difference was also statistically significant, as indicated by both a paired *t*-test, [*t*_(10)_ = 2.78, *p* = 0.019; *d* = 0.839].

**Table 12:** Leading characteristics during the highest SYN, lowest NSYN, highest and lowest MOI segments.

Dyad	Highest SYN	Lead in highest SYN	Lowest NSYN	Lead in lowest NSYN	Highest MOI HR synchrony	Lead in highest MOI HR synchrony	Lowest MOI	Lead in lowest MOI HR synchrony
P018	1.1423	Patient	0.0437	Therapist	0.5236	Therapist	0.2558	Therapist
P019	0.7503	Patient	0.0656	Therapist	0.3266	Patient	0.1904	Therapist
P025	0.9513	Patient	0.0376	Therapist	0.4403	Therapist	0.1659	Therapist
P028	1.3628	Patient	0.0532	Therapist	0.7650	Therapist	0.1487	Patient
P031	1.1503	Therapist	0.0164	Patient	0.5897	Patient	0.1726	Therapist
P032	0.5809	Patient	0.0637	Therapist	0.4721	Therapist	0.1478	Therapist
P035	0.8500	Patient	0.0656	Patient	0.3489	Patient	0.1395	Therapist
P037	0.5012	Patient	0.0671	Patient	0.2783	Therapist	0.1791	Patient
P047	1.0228	Therapist	0.0428	Patient	0.8553	Therapist	0.1445	Patient
P048	1.0701	Therapist	0.0716	Therapist	0.4166	Patient	0.2093	Therapist
P057	1.3203	Therapist	0.0392	Therapist	0.3428	Therapist	0.1209	Therapist

**Table 13:** Contingency table: HR synchrony leading and segment type.

HR synchrony leading	Segment type		
	SYN	NSYN	Total
Patient	29	17	46
Therapist	15	27	42
Total	44	44	88

**Table 14:** Chi-square and Fisher's exact test to confirm the association of HR synchrony leading and segment type.

Measure	Value	*df*	*p*
*χ^2^* tests	6.56	1	0.010
Fisher's exact test			0.018
*N*	88		

More MOI segments were therapist led than patient led (27:23), therapist leading (7:4) was predominant in both MOI segments with the highest HR synchrony and in MOI segments with the lowest HR synchrony (8:3; Table 9). There was no statistical difference in leading across all MOI segments and no associations were found between the highest and lowest MOI HR synchrony and the leading.

## 5 Discussion

### 5.1 Presence of dyadic heart-rate synchrony during MT intervention

Dyadic HR synchrony during MT intervention was significantly above the pseudo HR synchrony with a medium effect size. To the best of our knowledge, this was the first study to demonstrate the phenomenon of HR synchrony beyond coincidence in an MT clinical setting, thus supporting our primary hypothesis. We also found that the HR synchrony, patient leading component was more important than the therapist leading component. Neugebauer and Aldrige's study in 1998 involved a physicist visually examining the plots of the heart rates, and the study by (Gordon et al. 2020) demonstrated in a non-MT setting that inter-beat interval synchrony was significantly higher during drumming than at rest. Based on the interpretation of HR synchrony in psychotherapy research, it is reasonable to conclude that HR synchrony during MT intervention indicated a greater focus on the patient (Palumbo et al., 2017) and could have a positive impact on the session-level outcomes (Atzil-Slonim et al., 2023). The presence of high HR synchrony especially patient leading synchrony may also mean that the music therapist demonstrated empathic behaviors (Kodama et al., 2018; Neugebauer and Aldridge, 1998) and that there was shared experience (Mayo and Gordon, 2020) and the MT intervention was a meaningful encounter (Conroy et al., 2022).

### 5.2 Moments of interest exist not only during high heart-rate synchrony

Although the group's average MOI HR synchrony value (0.3229) was higher than the intervention average HR synchrony (0.3012), on the individual dyad level, only 4 of the 11 dyads' average HR synchrony during MOI segments was higher than their average intervention HR synchrony. On the segment level, 48% of MOI segments' HR synchrony was higher than the corresponding intervention HR synchrony, and 52% were lower. A detailed breakdown of the MOI segments of these 11 sessions, in terms of time (seconds), demonstrated only a slight tendency to overlap with SYN segments, at 53.65%, compared to 46.35% overlapping with NSYN segments. This indicates that the therapist's selection of MOI segments was not steered toward high HR synchrony and that from the perspective of therapeutic process, NSYN segments should not be dismissed as irrelevant. NSYN moments appeared to be equally important and crucial to the therapy process, aligning with the current literature. According to (Gordon et al. 2025), synchrony in a dynamical system, such as a social interaction, involves a perpetual coming in and out of synchrony. These authors warned that relying only on high synchrony as a measure would under-utilize the dynamical measures of synchrony (Gordon et al., 2025). In addition, successful interpersonal coordination may require dyads to move toward and away from each other dynamically, as these dynamics are complementary and adaptive (Mayo and Gordon, 2020; Feldman, 2021).

It is interesting to note that 27 of the 50 MOI segments overlapped with SYN or NSYN segments, which lasted only 30 s each, considering that the total duration of the MT interventions lasted 281.81 min. This could suggest a connection between MOI and the very high or very low physiological synchronization of HR, and the manifestation of the dyadic interaction was detectable physiologically during MOI, just as the study from (Neugebauer and Aldridge 1998) suggested that the patterns of cardiovascular behavior and interaction were structurally isomorphic.

### 5.3 High heart-rate synchrony segments were patient-led

A significant statistical difference was demonstrated between patient leading and pseudo patient leading in HR synchrony, but therapist leading did not. Looking at the level of the entire duration of the MT intervention, there was no significant difference between therapist-led and patient-led. This was however, not the case when we analyzed the four highest (SYN) and the four lowest (NSYN) synchrony segments. Our results showed a significant association between HR synchrony leading and the segment type, i.e., SYN or NSYN. HR synchrony during SYN segments was more frequently led by patients, while HR synchrony during NSYN segments was more commonly led by therapists. Within the dyad, there was also a statistically significant difference between the patient leading in SYN and the patient leading in NSYN. These findings suggest that HR synchrony is significantly associated with HR synchrony leading, with patient-led moments being more likely to coincide with higher synchrony segments.

The significance of leading during synchrony is considerable for the association between nonverbal synchrony and therapeutic success (Altmann et al., 2020). It is, nonetheless, difficult to interpret HR synchrony leading and its meaning across contexts because of a lack of reference literature. Considering that cardiac synchronization cannot be voluntarily steered (Kodama et al., 2018; Erdoes and Ramseyer, 2021) and given the observation that patterns of cardiovascular behavior and corresponding dyadic interactions suggest structurally isomorphic shapings (Neugebauer and Aldridge, 1998), patient leading could mean that patients were driving the interactions during the highest SYN segments, and the therapist was driving the interactions during the lowest NSYN segments. In the study by (Asher et al. 2021), patients with social anxiety turned their attention inward during social situations, hindering synchronization with external cues and introducing changes in heart rate that others have difficulty synchronizing with (Asher et al., 2021). Therapist leading in HR synchrony could indicate that the therapist was attempting to direct, advise or lead the patient out of the situation (Kodama et al., 2018) or was waiting ahead for the patient to join (Schmid, 2014). To shed more light on the possible meaning of HR synchrony leading, the videos of the MT intervention in this study should be analyzed on the interactional behavioral level.

### 5.4 The elusive construct of therapy readiness

The patient's self-reported therapy readiness had no significant associations with intervention HR synchrony. However, the therapist's assessment of the patient's therapy readiness was negatively associated with the HR synchrony [*F*_(1, 9)_ = 5.82, *p* = 0.039], with an *R*^2^ = 0.393, indicating that therapist assessment explained 39.3% of the variance in HR synchrony. This implies that there will be higher heart-rate synchrony when the therapist assesses a patient as being unready for therapy. We had previously found that low patient self-reported therapy readiness was associated with high NV synchrony (Yap et al., 2022). We postulate that it may be possible for the music therapist to sense the underlying unreadiness, and the more she felt that the patient was unready, the greater her intention to attune and guide the patient. This argument agrees with the proposed humanistic view of entrainment in neurorehabilitation (Sena Moore and LaGasse, 2018) and with the suggestion on intentionality and synchrony (Gordon et al., 2020). However, as the patient's self-reported therapy readiness had no significant associations with intervention HR synchrony, this study cannot confidently conclude the relationship between therapy readiness and HR synchrony, thus it remains a conjecture. It had been challenging to assess the association between the therapy readiness concept and synchrony. Not only is the concept of therapy readiness subjective, but it may also fluctuate throughout the session. This study assessed therapy readiness at the very beginning of the session, but the perceptions of therapy readiness from both parties might have changed after coming into contact. Future studies could explore this in more detail, also using a bigger cohort and reporting the initial therapy readiness at the end of the session.

### 5.5 Associations between nonverbal synchrony and heart-rate synchrony

The patient's self-reported therapy readiness was negatively associated with dyadic posttalk NV synchrony. The therapist's assessment of the patient's therapy readiness was negatively associated with dyadic HR synchrony during MT intervention. We also found no association between NV synchrony and HR synchrony within our sample. This was unsurprising as current literature has also highlighted the inconsistent relationship. The phenomenon of interpersonal synchrony (e.g., HR and NV synchrony) is recognized by some researchers as inherently nonlinear (Gordon et al., 2020), as it arises from dynamic interactions within a complex system; a change in one part of the system does not lead to a proportional effect (Demos and Palmer, 2023). It is also often non-stationary as it varies over time. The method used in this study, i.e., windowed cross-correlation, addresses the issue of non-stationarity by allowing synchrony to vary across time. It is also considered a robust method used in many research studies on interpersonal synchrony (Palumbo et al., 2017). However, it does not address nonlinear dependencies; thus, behavioral and HR synchrony could have contributed to measured outcomes independently over time, and their alignment might not have been captured (Gordon et al., 2020). Previous studies had warned against assuming a direct link between different synchrony modalities (Palumbo et al., 2017; Gordon et al., 2020). In the meantime, the adaptive systems model suggests that high levels of both types of synchronies may indicate a more adaptive system (Mayo and Gordon, 2020), and an increase in the connection between synchrony patterns from different modalities would be greater when there is a pull to synchronize (Gordon et al., 2025). A former study measured NV synchrony before and after the MT intervention (Yap et al., 2022), while HR synchrony was measured during it. Thus, understanding the lack of associations is complex. However, exploring dyadic interactions during significant decreases vs. increases in NV synchrony post-MT intervention could have promising potential.

### 5.6 Clinical considerations for music therapy intervention duration

This study revealed a statistically significant positive correlation between the duration of MT intervention and HR synchrony, the highest HR synchrony values, as well as a significant negative correlation with the lowest HR synchrony value. Grouping the HR synchrony values into blocks of 5 min showed that the optimal MT intervention duration for high HR synchrony values was 20–25 min for this sample. This block had the highest mean HR synchrony value, the highest HR synchrony value and the most significant number of the top five per cent of the HR synchrony values. Furthermore, dyads with intervention HR synchrony above the 75th percentile had MT interventions lasting at least 25 min. Although the subject of optimal duration of the MT session has not been firmly established (Braun Janzen et al., 2022), several studies have proposed that a duration between 20 and 30 min of active intervention (Ghai et al., 2018; Ghai and Ghai, 2019; Ruotsalainen et al., 2022) is optimal for therapy. Our results suggest that MT interventions of about 25 min could yield higher HR synchrony. MT interventions below 25 min could be less effective if a high HR synchrony is desirable. For an MT intervention to be at least 25 min long, the entire MT session would take about 60 min to accommodate 15 to 20 min of pretalk and posttalk and time for the necessary preparation and documentation. Even though the sample size of the present study is small, our findings on the role of MT intervention duration contribute valuable quantitative data to the existing knowledge, as it is already known that one of the main factors in stroke rehabilitation that hinders the development of these therapeutic alliance-building factors is the lack of time (Heredia-Callejón et al., 2023). Clinicians face constant pressure to shorten therapy duration due to hospital policies or healthcare economics in many parts of the world. The results suggest a potential risk of reduced efficacy from such measures, which may prolong patients' recovery time. This study could bring more awareness to the importance of MT intervention duration and encourage more research into this urgent issue.

### 5.7 Strengths and limitations

The results of this study should be interpreted with caution due to the limited sample size. The small sample size reduces the generalizability of the findings and makes it less likely to identify significant correlations. A bigger cohort could provide greater clarity to future investigations, particularly regarding the duration of MT intervention. In this study, three of the 11 dyads had less than 20 min of MT intervention. The results of this study are nevertheless meaningful; research on synchrony of physiological processes using multimodal data is rare and even less likely in an authentic clinical setting.

Specific exclusion criteria meant that patients were not included from the entire spectrum of neurological disorders, raising further concerns for the generalizability of the results of this study. However, this study has provided unique opportunities to identify the existence of NV synchrony previously and HR synchrony, which inferred a therapeutic relationship and alliance. Future publications on therapeutic interactions during HR synchrony and MOI have the potential to provide insight into the hidden physiological phenomenon of heart-rate synchrony.

MOI segment selection could have been more robust by inviting patients to view and co-select the material (Tucek et al., 2022). In this present study, due to the already extensive patient burden in data collection for the overarching project, the patients were not asked to select the MOI segments.

Efforts were made to reduce bias by ensuring the MOIs were selected before calculating HR synchrony. Moreover, even though they were chosen solely by the music therapist/researcher, the selected MOI segments had not shown a tendency toward high heart-rate synchrony.

The dual role of the music therapist and researcher could have also introduced bias. However, the dual role could also be recognized as dual quality, as the researcher also had prior knowledge of the therapeutic relationship.

This study did not capture the representation of the low end of the scale of therapy readiness. Patients who participated in this study were highly motivated, but as MT was non-compulsory, patients who were not motivated for MT would simply not participate. Furthermore, therapy readiness was not only a non-objectifiable concept; it was assessed at the very beginning of the session, which could have fluctuated during the session. Future investigations may consider asking the initial therapy readiness retrospectively at the end of the session, with an add-on question to indicate if it had changed. These factors could have contributed to the lack of correlation between patients' self-reported therapy readiness and the synchronies.

The use of windowed cross-correlation method is limited in its ability to capture the nonlinear dynamics fully. However, it is a widely applied and accepted method that accounts for the non-stationarity of synchrony and allows the exploration of fluctuations across time.

## 6 Conclusion and future directions

This study has shown that during MT intervention, HR synchrony exists beyond chance; critical moments of the therapy process can occur during the four highest or lowest HR synchrony, and the therapist may perceive these changes in HR synchrony.

Future studies should aim for a larger sample using the simple clinical protocol. This would facilitate the creation of sub-samples matched on key factors, such as duration and type of MT intervention. Additionally, researchers should investigate the influence of different segment sizes and lags, as well as the use of absolute (as in the present analyses) or non-absolute cross-correlations. The pseudo-HR synchrony could also be constructed using the corresponding data of the 5 min block for comparative analysis. Future investigations should delve deeper into the leading characteristics of NV and HR synchrony, thereby enhancing the interpretation of their meaning. Finally, additional clinical studies using a multimodal approach could validate this study's findings and further our understanding of how the different modalities relate to one another.

The computational methods have made it possible to visually perceive HR synchrony. These hidden physiological changes between two people were made accessible, and the heart in this narrative could have implications for rehabilitation policies. The finding of an association between duration of MT intervention and the level of HR synchrony could be one of them, as institutions should be cautious about reducing therapy time or replacing it with other responsibilities like documentation and preparation. Nevertheless, the importance of HR synchrony in MT in neurorehabilitation still needs evidence of its desirability. Qualitative video content analysis of the dyadic interaction during MT intervention could demonstrate its desirability and with that, we may be able to uncover more of the potential of this hidden gem.

## Data Availability

The raw data supporting the conclusions of this article will be made available by the authors, without undue reservation.
