# Supplementary material for: It takes time to synchronize: the emergence of dyadic heart-rate synchrony during music therapy in neurorehabilitation
Source: Front Psychol. 2026 Apr 17;17:1629778. doi: 10.3389/fpsyg.2026.1629778 (PMC13133023; doi:10.3389/fpsyg.2026.1629778)
Supplement: Supplementary file 1 [file Data_Sheet_1.pdf]

Appendix I: Example of visual inspection of the filter procedure

The raw R-R data were uploaded into Kubios HRV Scientific 4.2.0, and the period of music intervention was selected; in this example, it lasted 29 minutes and 45 seconds. The top image 1) Raw-R-R shows an artefact. The middle image shows the same artefact zoomed in on the Kubios program. The bottom image 3) After-Filter, shows the correction by the Autonom Platform.

1) Raw R-R

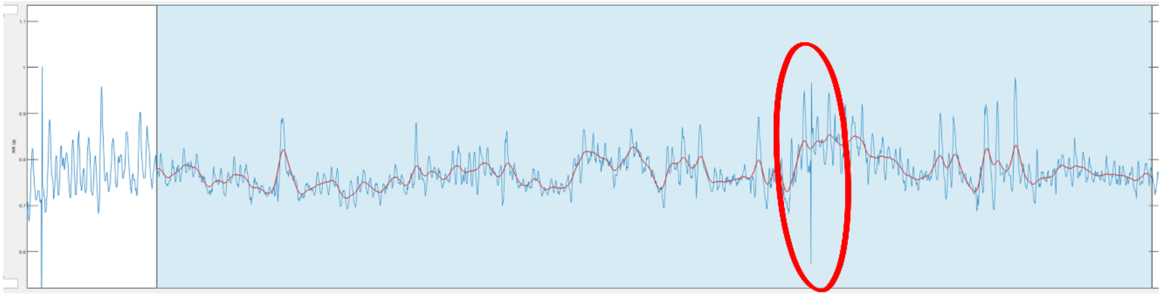

2) Close-up

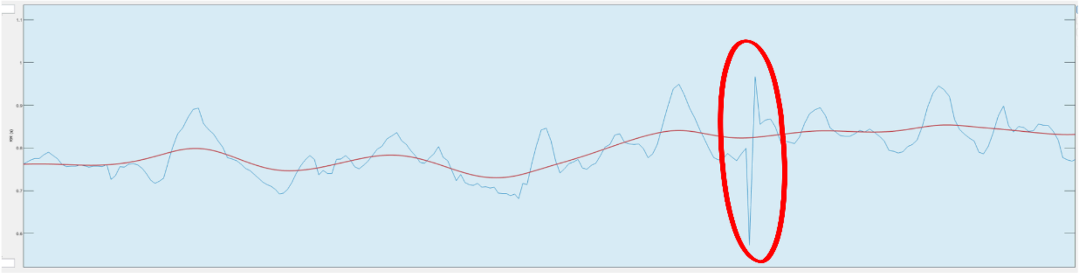

3) After- Filter

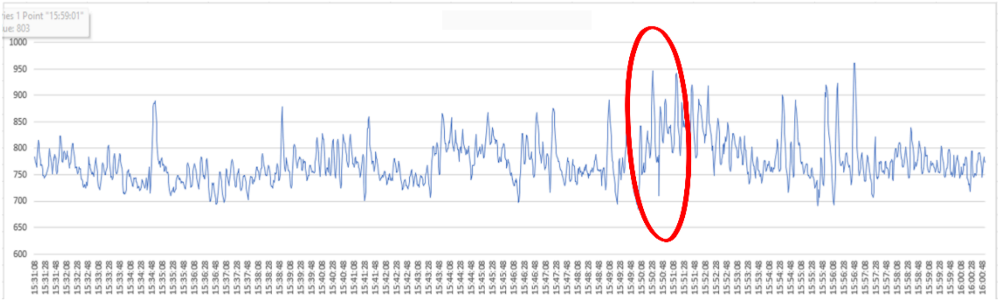

Appendix II: Heart rate data during music therapy interventions

HR patients											
	PO18	PO19	PO25	PO28	PO31	PO32	PO35	PO37	PO47	PO48	PO57
Mean	77.70	69.00	78.80	66.80	64.70	81.30	69.50	78.10	83.60	78.90	65.00
Median	78.10	68.70	79.00	66.30	63.80	81.30	68.70	77.40	82.60	78.40	64.80
Standard deviation	3.90	3.62	4.63	3.46	4.93	4.66	4.01	5.68	6.04	3.83	4.21
Minimum	62.40	60.90	66.10	58.80	51.20	67.10	60.90	65.90	66.20	69.10	52.50
Maximum	87.60	82.20	93.80	82.10	85.60	96.80	90.50	106.00	111.00	110.00	81.10
